# ASO Author Reflections: Variation in Hospital Mortality After Complex Cancer Surgery: Patient, Volume, Hospital, or Social Determinants?

**DOI:** 10.1245/s10434-024-14898-6

**Published:** 2024-01-11

**Authors:** Muhammad Musaab Munir, Timothy M. Pawlik

**Affiliations:** Department of Surgery, The Ohio State University Wexner Medical Center and James Comprehensive Cancer Center, Columbus, OH USA

## Past

Access to high-quality surgical services for cancer patients is increasingly hindered by a diverse array of obstacles. Key factors, such as patient attributes (i.e., age, sex, and race), hospital traits (i.e., teaching status and the ratio of nurses to patients), surgical case volume, and social determinants (i.e., insurance status, travel time to the hospital, and social vulnerability index), have been identified as impediments to the provision of high-quality surgical care.^1,2^ Recent healthcare technology and infrastructure improvements have concentrated high-risk cancer surgery in specialized centers, which, while potentially improving postoperative outcomes for some, raises concerns that excessive centralization may exacerbate inequities and reduce access for marginalized groups.^3^ The association between surgical volume and perioperative outcomes does not exist in a vacuum, and the effects of various other factors at the patient, hospital, and neighborhood levels on this relationship remain ill-studied. As such, the current work sought to define the contributions of patient characteristics (PC), hospital characteristics (HC), case volume (CV), and social determinants (SDoH) on variations of in-hospital mortality (IHM) among patients receiving complex cancer care within the state of California.^4^

## Present

A total of 52,838 patients underwent cancer surgery (esophagectomy [ES]: *n* = 2700, 5.1%; pneumonectomy [PN]: *n* = 30,822, 58.3%; pancreatectomy [PD]: *n* = 7530, 14.3%; proctectomy [PR]: *n* = 11,786, 22.3%) across 294 hospitals. The IHM for the overall cohort was 1.7% and varied from 4.4% for ES to 0.8% for PR. On multivariable regression, PC contributed the most to the variance in IHM (overall: 32.0%, ES: 21.6%, PN: 28.0%, PD: 20.3%, PR: 39.9%). Among the overall cohort, CV contributed 2.4%, HC contributed 1.3%, and SDoH contributed 1.2% to the variation in IHM. CV was the second highest contributor to IHM among ES (5.3%), PN (5.3%), and PD (5.9%); however, HC was a more important contributor among patients who underwent PR (8.0%). The unexplained variance in IHM was highest among ES (72.4%) followed by PD (67.5%) and PN (64.6%) patient groups. These data serve to highlight that much of the variability in IHM was due to patient-related factors rather than case volume, hospital characteristics, and social determinants. In turn, more than one-half of the variation in postoperative outcomes remains unexplained for a large subset of patients with complex cancer.

## Future

Results from the present study demonstrate the importance of patient optimization and risk identification, stratification, and mitigation to improve the quality of oncologic surgical care. Moreover, these findings emphasize that applying fixed hospital case volume thresholds for quality improvement, as seen in initiatives, such as the Volume Pledge, risks worsening healthcare access disparities, and overlooks factors, such as patient preferences geospatial access barriers and patterns of insurance referrals.^1,5^ This focus on case numbers, despite its proven association with better outcomes, may neglect a more comprehensive approach to healthcare improvement. Enhancing healthcare equitably may require prioritizing patient rehabilitation, upgrading hospital resources, and engaging underprivileged communities. Addressing unexplained variances in IHM is crucial and likely tied to complexities in continuous quality improvement (CQI) initiatives and hospital safety cultures, which are not easily tracked in administrative databases. Analyzing these aspects may require detailed clinical data analysis, utilization of advanced AI techniques, such as natural language processing and machine learning, as well as more sophisticated statistical methods, such as mediation analysis.^6,7^

## References

[1] Munir MM, Endo Y, Alaimo L (2023). Impact of community privilege on access to care among patients following complex cancer surgery. Ann Surg.

[2] Ellis L, Canchola AJ, Spiegel D, Ladabaum U, Haile R, Gomez SL (2018). Racial and ethnic disparities in cancer survival: the contribution of tumor, sociodemographic, institutional, and neighborhood characteristics. J Clin Oncol.

[3] Munir MM, Endo Y, Woldesenbet S (2023). Variations in travel patterns affect regionalization of complex cancer surgery in California. Ann Surg Oncol.

[4] Munir MM, Woldesenbet S, Endo Y (2024). Variation in hospital mortality after complex cancer surgery: Patient, volume, hospital or social determinants?. Ann Surg Oncol.

[5] Gani F, Azoulay D, Pawlik TM (2017). Evaluating trends in the volume-outcomes relationship following liver surgery: Does regionalization benefit all patients the same?. J Gastrointest Surg Off J Soc Surg Aliment Tract.

[6] Barber EL, Garg R, Persenaire C, Simon M (2021). Natural language processing with machine learning to predict outcomes after ovarian cancer surgery. Gynecol Oncol.

[7] Woldesenbet S, Munir MM, Pawlik TM (2023). Invited commentary: mediation analysis and cancer screening, identifying areas to target to improve cancer disparities. J Surg Oncol.

